# COVID-19 challenges, responses, and resilience among rural Black women: a study protocol

**DOI:** 10.3389/fpubh.2023.1156717

**Published:** 2023-06-02

**Authors:** Shan Qiao, Sara Wilcox, Bankole Olatosi, Xiaoming Li

**Affiliations:** ^1^Department of Health Promotion Education and Behavior, Arnold School of Public Health, University of South Carolina, Columbia, SC, United States; ^2^South Carolina SmartState Center for Healthcare Quality, University of South Carolina, Columbia, SC, United States; ^3^Department of Exercise Science, Arnold School of Public Health, University of South Carolina, Columbia, SC, United States; ^4^Prevention Research Center, University of South Carolina, Columbia, SC, United States; ^5^Department of Health System Policy and Management, Arnold School of Public Health, University of South Carolina, Columbia, SC, United States

**Keywords:** COIVD-19, Black or African American, women, rural health, psychological resilience, community-based participatory research

## Abstract

Despite the aggregated burdens and challenges experienced by rural Black women during the COVID-19 pandemic, many likely also demonstrated strength and resilience to overcome challenges. A mixed methodology and a community-based participatory approach will be used to collect multilevel data on challenges, responses, resilience, and lessons during the pandemic from Black women, community health workers, and community leaders in rural areas in South Carolina (SC). Specifically, the unique circumstances and lived experiences of rural Black women during the COVID-19 pandemic will be documented to understand their needs regarding effective management of social, physical, and mental health challenges through focus group discussions and in-depth interviews with Black women, community health workers, and local community leaders recruited from rural SC communities. Barriers, facilitators, and potential impacts of multilevel resilience development will be identified through a survey administered among rural Black women recruited from 11 rural counties (with one as site for a pilot testing of the questionnaire). A report for public health practice will be developed, including recommended strategies to optimize health systems' emergency preparedness and responses through triangulation of qualitative and quantitative data from multiple sources. Findings in the proposed study will provide valuable references in terms of addressing social determinants of health factor challenges during the pandemic, fostering resilience, and informing evidence-based decision-making for policymakers. The study will contribute to the development of public health emergency preparedness plans, which can promote the resilience of women, their families, and local communities as well as optimize effective preparedness and response of health systems for rural Black women and their families during infectious disease outbreaks and other public health emergencies.

## Introduction

Existing literature suggests that women are more likely to bear the brunt of socioeconomic and health consequences of the pandemic due to the compounded effect of pre-existing gender inequity, their social role as caregivers, work-life conflicts, increased domestic violence, and limited access to healthcare services in the context of COVID-19 (1–4). Unpaid caregiving during the pandemic has imposed a disproportionate burden on women who are often the primary caregivers for children and older adults (5). Lack of paid leave, family caregiving responsibilities, and traditional gender roles have placed additional strains on work-life conflicts (6, 7). When social isolation and distancing practices are being enforced in a pandemic, the risk of violence against women increases (8, 9). The pandemic also has impacted the availability and utilization of women's healthcare services such as sexual and reproductive health services and preventive care such as mammography screening (10, 11). Mental health issues for women related to financial and other stressors are also evident during the pandemic (12). Furthermore, national polls indicate that women are more likely than men to report negative mental health effects from worrying about COVID-19 (13, 14).

Black women from rural households in southern states are especially vulnerable, given the existing disparities in social determinants of health (SDOH), health infrastructure, and access to healthcare resources (15–18). The disproportionate effects of COVID-19 among racial/ethnic minority groups were present from the beginning of the pandemic (19). Counties with large Black populations experienced greater case, mortality, and progression rates of the disease than counties with small Black populations (20). Analysis of data from the COVID Tracking Project highlighted that the national COVID-19-related mortality rate for Black people was 2.4 times higher than that of White people (21). The COVID-19 pandemic further elevated social disparities. Black people were more likely than White people to experience job or wage loss because of the pandemic (44 and 38%, respectively) (22) and experienced higher levels of food insufficiency and rent or mortgage defaults relative to other racial and ethnic groups as well (23). In addition, the southern United States is a region in which structural racism and oppression have resulted in poor health infrastructure, limited access to care (e.g., lack of health insurance and geographic maldistribution of healthcare services) (24), and biased and suboptimal care (25, 26).

Despite the aggregated burdens and challenges experienced by rural Black women during the pandemic, it is likely that many have also demonstrated strength and resilience to overcome challenges and manage critical resources for themselves and their families (27, 28). Resilience refers to the capacity to which individuals are able to respond to stress-induced challenges and burdens (29). Within the literature over the past few decades, there has been a shift to consider resilience not only as an individual trait but, from the ecologic perspective, an bidirectional interaction between individuals and their environment (30). Thus, resilience can now be understood as a multidimensional, responsive, and dynamic process across the life span (31). Resilience can be cultivated through a series of protective factors such as social support, self-efficacy, positive self-perception, and an optimistic perspective of the future (32–34). The extant literature demonstrates the essential role of protective factors in aiding in the bolstering and maintenance of resilience within the individual, community, and the institutional levels of the socioecological model in times of crisis including public emergencies (35–39). Several recent studies particularly examined the resilience model for older people in the context of COVID-19 pandemic (40, 41).

Resilience can play a significant role in the coping and responses to the pandemic (42, 43). However, empirical data are especially limited regarding the needs and challenges among Black women in rural areas, how they successfully cope with SDOH challenges during the pandemic, and the facilitators of resilience from multiple levels (e.g., family, community, policy) (14, 44–46). Furthermore, there are limited data on first-hand evidence from front-line health workers and key stakeholders in rural areas such as community health workers and community leaders who are rooted in local communities, serve women in rural areas through connecting local neighborhood and external resources, and can provide insights and recommendations to public health policy and practice (47).

To address these gaps, a mixed-methods exploratory study will be conducted to collect multilevel data on challenges, responses, and resilience among rural Black women during the pandemic. A community-based participatory approach will be used to engage Black women, community health workers, and community leaders in South Carolina (SC), a state with 27% of its population being Black and 33.7% of its population living in rural areas (48). The advanced understanding of the resilience process and facilitators and barriers of resilience for women will contribute to optimizing emergency preparedness and response for special needs and challenges identified for Black women from rural areas and their families (42).

## Methods and analysis

### Research setting and community engagement

#### Research setting

SC is a largely rural state (48), currently ranked the 42nd overall healthiest state, 49th for cost of care, and 32nd for access to care in the nation (49). A majority of counties in SC (45 of 46) are designated as Health Professional Shortage Areas (50, 51). SC also ranked at the bottom for various health outcome indicators in 2019: 49th in infectious diseases, 41st in maternal mortality, and 39th in infant mortality (52). According to the most recent American Community Survey, White people account for 63.7% of the whole population, followed by Black people (27.03%), and Hispanics or Latinos (6%). In SC, racial disparities exist in many healthcare outcomes such as breast cancer (53, 54), stroke (55), maternal health (56, 57), and cervical cancer (58, 59). Considering SC's poor health ranking, striking disparities in many health outcomes, racially diverse population, and historical Southern context, SC has an appropriate environment to explore and understand lived experiences of Black women living in rural areas facing SDOH challenges during the pandemic. The study will be conducted in 11 rural counties (out of totally 46 counties in SC) in which Black people account for over 30% of the total population. These counties have been heavily hit by COVID-19. The participants of the study are adults with a large range of age in order to explore the lived experiences across life course.

#### Community engagement and Community Advisory Board

The research team will closely work with community-based organization and community health workers on the study design and implementation. Our main local partner is the South Carolina Community Health Worker Association (SCCHWA), a community-based organization made up of community health workers and their supporters in SC. It provides a forum for networking and sharing of strategies and resources as well as a foundation for education and training of community health workers. The SCCHWA has implemented numerous health promotion education projects with local partners across SC, including the multilevel COVID-19 vaccination promotion project among Black communities with collaboration from our team. With the assistance of the SCCHWA, a CAB will be assembled to include Black women, community leaders, government officers, healthcare providers and community health workers. The CAB members will either live in or serve people in the local communities or have strong connections with rural communities. The CAB will meet every 2 months to provide advice regarding community engagement, study protocol development, and research implementation and dissemination.

### Study design

The proposed mixed methodology study consists of three main specific research phases in term of study design. Phase 1 is qualitative research via focus group discussions (FGDs) and in-depth interviews with Black women, community health workers and community leaders in rural areas. Phase 2 is quantitative research including the adaption of assessment tools and implementation of a survey among Black women from the research sites. Phase 3 includes data triangulation and report writing. A community charrette approach (60) will be applied in report revision and finalization to empower local partners.

### Qualitative research

#### Focus group discussions

FGDs will be conducted with Black women, community health workers, and local community leaders recruited from various rural SC communities to document the unique circumstances and lived experiences of rural Black women during the COVID-19 pandemic and to understand their needs regarding effective management of social, physical, and mental health challenges.

FGD is selected to collect qualitative data for Aim 1 since it is a time-efficient and interactive approach to conduct need assessments among diverse subgroups (61). With the assistance of SCCHWA and the CAB, participants will be purposely recruited from the study sites including Black women (*n*−15), community health workers serving rural communities (*n*−10), and local community leaders (*n*−10) to conduct 3 FGDs. The Black women will include young adults (18–34 years of age), middle-aged adults (35–59 years of age), and older adults (≥60 years of age) given that COVID-19 may impose different challenges for women at different age. COVID-19 infection history of women and their families will also be considered to maximize the focus group representativity. Community health workers will include the ones staying short in the local communities (<3 years) and staying long (≥ 3 years). Community leaders may include people from churches, non-government organizations, grassroot organizations, or other trusted messengers in local communities with covering both health-related organizations and non-health-related organizations. Potential eligible participants will be identified through local community health workers in study sites (for Black women), SCCHWA staff (for community health workers), or recommendation by the CAB (for community leaders). Research staff at SCCHWA will conduct outreach and recruit the participants, highlighting that the FGDs are confidential.

To ensure that FGD participants have sufficient time and opportunities to express their opinions and share their experiences, the research team will hold relatively small FGDs with 5–6 participants per group. The group discussion guidelines will be drafted by the research team and then be reviewed and discussed by our local partners and the CAB to ensure that the questions are meaningful in local contexts and asked in appropriate way/language. The FGDs will be facilitated by experienced SCCHWA project staff, but research staff_will attend all of the FGDs as backups and for assistance. The main topics of the FGDs may include: (1) lived experiences in response to the COVID-19 pandemic, including COVID-19 prevention, testing, and treatment and health seeking; (2) challenges with various health and social aspects during the pandemic; (3) impacts of COVID-19 on physical health, mental health, family relationships, social networks, and socioeconomic conditions; (4) needs and available resources to address multiple challenges, especially SDOH challenges; and (5) unmet needs and additional resources they need to deal with the evolving pandemic and life recoveries. To avoid research burden of the participants, the research team will select relevant topics and tailor the questions to different groups. For example, for Black women participants, the questions will focus on their own experiences; for community leaders, the topics will also include their observations of the whole communities; and for community health workers, the discussion will focus on lessons in bridging communities and healthcare systems, reflections on organizational responses, and suggestions toward capacity building as public health front-line workers. The FGDs will last about 1 h and be held in a private conference room at the SCCHWA site offices. Considering the transportation cost and burden for participants, online FGDs via an Internet conference (e.g., Team, Zoom meetings) will also be prepared for, depending on local logistics as well as suggestions and preferences of the participants.

#### In-depth interviews

In-depth interviews will be conducted with Black women, community health workers, and community leaders recruited across the communities to explore effective strategies that women use in coping with various challenges in the pandemic and identify barriers to and facilitators of multiple resilience. Given that people will apply various coping strategies and demonstrated different types of resilience, in-depth interviews will be an appropriate approach to collecting qualitative data regarding our specific research aims, which will offer opportunities for one-on-one, in-depth conversations with minimum influence of others on the interviewee.

Following a similar study protocol as used in the FGDs, the research team will purposely recruit about 20 Black women living in rural areas, 10 community health workers, and 10 local community leaders for in-depth interviews. A “saturation” approach will be applied in the interviews, whereby respondents will be interviewed until a point that no significant new data are anticipated from additional interviews. Data saturation will be assessed after ~60% of the interviews have been conducted with each group of participants.

Separate interview guides will be developed for Black women, community health workers, and community leaders. Semi-structured qualitative interview guides will be created with significant input and guidance from the local CAB. The interview guides will be grounded in phenomenological and constructivist frameworks, which provide a general structure for discussion but require participants to provide their own conceptualizations of terms and phrases based on their life experiences. The interview with Black women will document their lived experiences and various coping strategies in response to SDOH challenges during the pandemic; identify components of multilevel resilience, interactions of different resilience, and barriers and facilitators for resilience; and needs, expectations, and suggestions for health systems' emergency responses tailored to their needs. Interviews with community health workers and community leaders will focus on community resilience and institutional/organizational resilience observed and experiences during the pandemic, local resources, community connections, and their reflections and insights on optimizing emergency response strategies in healthcare systems and local communities. Additional topics will be added as appropriate and as indicated by the CAB and findings from the FGDs. With appropriate consent, the interviews will be audio-recorded. Interviewers will take field notes during the interviews to serve as a complementary data source. The field notes will include interviewees' non-verbal responses and interviewers' observations or impressions regarding the conditions of the interviews. Each interview will take 1 h led by a trained interviewer in a private room. Online interview will be conducted if preferred by the participants.

#### Qualitative data analysis

The analysis of both FGD and in-depth interview data will be guided by grounded theory (62) in order to obtain key themes based on data itself rather than preexisting opinions. This inductive approach helps prevent preconceived notions from interfering with the data collection and analysis (62). In keeping with the grounded theory principles, data analysis will run concurrently alongside data generation. Transcription and coding will take place after the first three interviews for each group. The line-by-line open coding will sensitize us to the range of potential meanings in the data and identify themes. Axial coding will be used to elucidate relationships between themes and subthemes along with their properties and dimensions. Memo writing and diagramming will be used to develop themes and relationships between themes. Research staff will independently code all of the transcripts. Any coding disagreements will be resolved through discussions. Representative quotes will be selected verbatim to illustrate key findings. Data analysis will be conducted through the software NVivo 12. The project coordinator and research staff at SCCHWA and CAB members will also contribute to result interpretation and findings dissemination.

The findings from qualitative research in Phase 1 will be used to inform the cultural adaptation of assessment tools in Phase 2 and data triangulation and report development in Phase 3. Specifically, the measurement instruments and existing scales will be adapted regarding resilience, coping, and other psychosocial wellbeing outcomes in the local context. The results of the qualitative studies will advance our understanding of the social and cultural environment that surrounds Black women, their families, and community health workers, and thus assist us in measurement selection and adaptation. Reports will be developed on needs assessment and strategy recommendations based on the rich qualitative evidence. For example, the materials of lived experiences and challenges of Black women, community health workers, and other key stakeholders in rural communities as they have faced this public health crisis will inform potential interventions and policymaking in fostering resilience and readiness for public health emergencies among rural communities and healthcare systems. Specific scenarios and examples needed in the intervention will also be developed by extracting the qualitative data and/or citing representative quotes. From the perspective of the community-based research, our local partner will be engaged in each step of the study design and data collection and analysis, which will empower community health workers and increase their ownership of this project, and thus further strengthen the academic-community trust and collaboration.

### Quantitative research

#### Participants and recruitment

After discussion with our local partner SCCHWA, a cluster sampling approach will be used to recruit ~200 Black women living in rural areas in SC. Specifically, the research team will select 11 counties (with one county as the site for pilot testing) in SC as our study sites. About 20 Black women in each site will be recruited. With the coordination of research staff at SCCHWA, community health workers serving the rural communities in the study sites will recruit potential participants for the survey through disseminating project flyers at community activity centers, community clinics, grocery stores, and public libraries. Inclusion criteria include: (1) Black females; (2) at least 18 years of age; (3) living in the study site since the COVID-19 outbreak; and (4) not concurrently participating in any health promotion intervention. A half-day project training workshop will be conducted for the local research team (mainly composed of community health workers) in terms of study protocol, data collection, and research ethics. The trained research staff (survey interviewers) will confirm the eligibility of the participants; explain the study design, including the purpose, procedure, risk and benefit, and confidentiality issues; and invite them to participate. All who agree to participate will provide written informed consent.

#### Data collection

Survey interviewers (local community health workers who receive project training) will administer the survey to participants via Tablets. The Tablet will display and read (with a real human voice, utilizing a headset) the survey questionnaire in a private room (e.g., community health worker's office) in local counties where the participants are recruited. By using this method, the research team will not only ensure the privacy and quality of the data collection, but also ensure that varying degrees of literacy do not affect the individual's ability to understand the items. Clarifications or assistance (with the Tablet) will be provided on site by the interviewers as needed. It is estimated that the survey will take about 30 min. Participants will be instructed to take a short break (~5 min) after every 15 min as needed.

In the survey, basic screening will be conducted to avoid logic errors in completing the questionnaire. The project PI and local partner will take the responsibility of data quality control and monitoring during the data collection by randomly selecting and reviewing first five finished questionnaires and data record from each site. The questions and feedback will be provided to the research staff in a timely way through daily supervision by SCCHWA and regular meeting and monitoring by the USC research team.

#### Key measurements

The key measurements in this study are composed of primary outcomes, secondary outcomes, and individual background measures (to be collected through the survey questionnaire); and contextual measures (to be extracted from publicly available datasets). Most of the demographic and psychosocial and health behavior measurements used for Black women participants in this study are field-tested and validated in previous studies and have been shown to be reliable and valid. The measures will be further modified based on the specific aims of this study, qualitative study findings, and literature on resilience, coping strategies, mental health in the context of COVID-19. The final draft of all measures will be reviewed by the CAB and will be pilot-tested among 15 Black women recruited from pilot-testing site to obtain participants' perspectives on the clarity, cultural sensitivity, and appropriateness of relevant measures.

***Primary outcomes*** will be mental health symptoms measured by standardized self-reported scales with good psychometric characteristics (e.g., validity and reliability) in previous studies: (1) depression, measured Patient Health Questionnaire-9 (PHQ-9) (63). One recent literature review suggests solid evidence supporting the validity of the PHQ-9 as a unidimensional measure of depression. Used in major depressive disorder (MDD) screening with a cut-point of 11, its sensitivity was 95% and specificity was 88.3% (PPV 51.4%, NPV 48.6%) (64); (2) anxiety measured by Generalized Anxiety disorder-7 (GAD-7) (65). Confirmatory factor analyses suggest the 1-dimensional structure of the GAD-7 and its factorial invariance for gender and age. GAD-7 shows high reliability across gender and age groups (α = 0.89). Intercorrelations with the depression and the Rosenberg Self-Esteem Scale were *r* = 0.64 (*P* < 0.001) and *r* = −0.43 (*P* < 0.001), respectively (66). (3) Post-traumatic stress disorders, PTSD, measured by (PC-PTSD-5) (67). The PC-PTSD-5 is modified based on Primary Care PTSD screen (PC-PTSD) to reflect the new Diagnostic and Statistical Manual of Mental Disorders (DSM-5) criteria for PTSD. The PC-PTSD-5 demonstrated excellent diagnostic accuracy (AUC = 0.941; 95 % C.I.: 0.912–0.969) (68); and (4) domestic violence measured by a four-item scale that asks respondents how often their partner physically Hurt, Insulted, Threatened with harm, and Screamed at them. These four items make the acronym HITS (69). This is a short domestic violence screening tool widely used in a family practice setting. The sensitivity ranged from 30 to 100% and specificity ranged from 86 to 99%. The reliability is generally good (α ranged from 0.61 to 0.8) (70).

***Secondary outcomes*** will include multiple resilience and their resources: (1) individual resilience of Black women, for example, personal resilience strengths (71), coping strategies (72), self-concept (73); (2) family factors, for example, quality of relationship (74), and healthcare system factors, such as perceived acceptance and trust from healthcare facilities; (3) community resilience, for example, perceived social support (75); and (4) institutional resilience, for example, organizational resilience (an organization's ability to anticipate issues ahead of time and develop a plan for handling identified problems) (76).

***Individual background measures*** are basic sociodemographic variables, including: (1) age; (2) educational level; (3) marriage status; (4) household income; (5) health insurance; (6) employment; and (7) COVID-19 infection history, long COVID symptoms, or caregiving experience for family members or neighbors infected by COVID-19, if any.

***Contextual characteristics variables*** include aggregated county-level measures at the structural level, community level, and institutional level (Table 1): (1) Structural level: SDOH obtained from the American Community Survey (ACS) (77); (2) Community level: social capital data from an existing dataset from the county-level Social Capital Index Project in the US (78), behavioral and environmental risk exposure data obtained from Behavioral Risk Factor Surveillance System (BRFSS) and County Health Rankings & Roadmaps Program (CHRRP); and (3) Institutional level: health infrastructure data can be retrieved from Area Health Resources File (AHRF), including health professions capacity [primary care physicians (PCP) per 100,000 population, population to PCP ratio] and distance to health facilities. All the aggregated data are county-level measures so we control the cluster effect of various counties in the analysis.

**Table 1:** Contextual characteristics variables and their data sources.

**Variables at multiple socioecological levels**	**Data sources**
**Structural level**	
SDOH measures: population characteristics (race/ethnicity composition, urban/rural status), percent of population with a high school education, percent of population lacking health insurance, income inequality, median household income, percent of population unemployed, percent of population living in poverty, percent of population living with food insecurity, percent of population living in unstable housing	American Community Survey (ACS)
**Community level**	
Social capital index score: family unity (e.g., share of births in past year to women who were unmarried, share of own children living in a single-parent family), community health (numbers of non-religious, non-profit organization, religious congregation per 1,000 population and informal civic engagement), institutional health (Presidential election voting rate, mail-back census response rate, confidence in institution), collective efficacy (Violent crimes per 100,000 population)	Social Capital Index Project
Behavioral and environmental risk exposure: depression rate and poor mental health days, substance use indicators	Behavioral Risk Factor Surveillance System (BRFSS) County Health Rankings & Roadmaps Program (CHRRP)
**Institutional level**	
Health infrastructure: health professions capacity [e.g., primary care physicians (PCP) per 100,000 population, population to PCP ratio], health facilities capacity, distance to health facilities	Area Health Resources File (AHRF)

#### Data analysis

Given the preliminary nature of this work and the small sample size dictated by time and budget limitations of the 1-year research mechanism, quantitative analyses will focus on obtaining estimates of mental health outcomes and multilevel resilience among Black women living in rural areas and characteristics associated with these outcomes, for use in design of a larger study. Therefore, the specific analysis plan includes (1) Participant characteristics will be presented using counts and percentages for categorical variables and means and standard deviations or medians and inter-quartile ranges (IQR) for continuous variables; (2) Descriptive statistics will be used to evaluate distributions of the measures; (3) Psychometric characteristics of scales will be evaluated using Cronbach's alpha and factor analysis, then compared to published scale psychometrics in Black women if possible. The temporal stability of scales will also be investigated to ensure reliability. These approaches will help assess the utility of the instruments used for future analyses and research in this subpopulation; (4) Exploration and estimation of the associations between primary outcomes (mental health and domestic violence) and secondary outcomes (multilevel resilience), for which correlation analysis and ANOVA for continuous variables will be conducted; and (5) Potential cofounders (e.g., sociodemographic factors) will be evaluated for associations with outcomes using Wilcoxon rank sum or independent-sample *t*-tests, Spearman or Pearson correlations, and chi-square tests as appropriate. Multivariable analyses will be used to adjust for sociodemographic and other potential covariates (including aggregated county-level contextual characteristics). Cluster effect will be adjusted too in the regression analysis.

#### Power analysis

Since the proposed study is not a clinical trial or longitudinal study, it is not designed or powered to determine the overall intervention effect nor the causal relation between key variables. It is hard to calculate the power and appropriate sample size due to lack of information of key indicators. However, according to rule of thumb of the minimum sample sizes in absolute Ns, any *N* > 200 sample offers adequate statistical power for data analysis (79, 80). Therefore, the sample size of 200 in our quantitative study is still acceptable and the preliminary data analysis will help to provide some insights into the promise of a potential resilience-based intervention to inform a future RCT.

### Data triangulation and report development

#### Data triangulation

Different types of data will be synthesized and triangulated in different forms and from multiple sources, including inputs from our governmental and community partners and the CAB, findings from the qualitative research and quantitative research, published peer-reviewed and gray literature, conference presentations, government reports, and unpublished data. The data triangulation activities will engage various community and health organization stakeholders (e.g., through local data sharing and interpretation forums). The main results/themes will be cataloged using data-plotting worksheets to identify areas of convergence (“syntheses”) or divergence of the study findings from different sources of data (81). For issues with significant divergence from multiple data sources, the CAB and other key stakeholders will be consulted with for further clarification and interpretation. For results that remain inconclusive, the research team will generate research questions or hypotheses for future research.

#### Drafting the final report

With assistance from SCCHWA and CAB, the University of South Carolina (USC) research team will draft the final report on policy recommendations based on the outputs of data triangulation. Generally, five key issues will be covered in the policy recommendation report: (1) **Risk and vulnerability** including the key challenges, especially SDOH challenges among Black women in rural communities during the COVID-19 pandemic and their unique needs in healthcare access and mental health intervention; (2) **Resilience** including the manifestations of multilevel resilience (individual-, community-, institutional-level) extracted from participants' lived experiences and their coping strategies. (3) **Resources** including available resources for Black women and community health workers in local communities in response to public health emergencies as well as the types of resources they need but that are not yet available to optimize emergency responses; (4) **Community connectedness**. Rural communities could be connected with each other and shared resources through statewide health organizations such as SCCHWA. The practice will be discussed regarding resource sharing and collaborations across communities but within a common healthcare system; and (5) **Planning and procedures**. Recommendations will be provided about how to improve preparedness and readiness in response to public health emergencies through highlighting the take-home messages for policymakers in healthcare systems. The report will be tailored for community health workers as part of toolkit of their resilience development to optimize emergency preparedness and responses in research translation phase.

#### Finalizing report via community charrette

The report draft will be adapted and finalized through a community charrette among the CAB members. As a community engagement strategy recommended by the National Minority AIDS Council, a charrette is a collaborative planning process that purposefully brings together the expertise of community and academic research partners in order to strengthen partnerships, engage stakeholders, and make decisions regarding translational research (82). Since the launch of the charrette model in 2009 [i.e., originating from a clinical translational science award (CTSA) initiative], this process has been used successfully to launch community-engaged research initiatives across the clinical-translation spectrum (82, 83). This approach can help address specific community problems and provide a context for integrating design and scientific inquiries with local community knowledge (84).

The community charrette will be held in a USC or SCCHWA conference room to assure privacy or conducted in a Zoom platform using the “breakout discussion room” function, depending on the logistics and the COVID-19 situation at the time. CAB members will receive the report draft 2 weeks prior to the charrette and be required to review and provide feedback on its content and structure. The charrette will begin with a review of the charrette goals and an explanation of the procedures for the day. Participants (about 10–15) will be divided into groups of 3–4, and members of the research team will co-lead each of the small group discussions. Each group will discuss the same set of questions that are based on the charrette objective (e.g., feedback on each chapter, strengths and weakness, additional content, etc.), and a co-leader will record the primary points on poster paper. After completing small group discussions, the full group will re-convene, and a representative from each group will present their findings; other members will ask questions and points of clarification, and additional information will be added to the poster paper if needed. The poster paper notes become the primary data source. Field notes will be taken during the course of the charrette by two research staff, with observational and interpretive elements. At the end of the charrette, the CAB will engage in a process of critical reflection regarding the group and develop combined reflection notes based on these conversations.

The report will be further revised and finalized based on the data/notes collected from the community charrette among the CAB members. The USC research team will lead the revision and hold multiple meetings of research staff (from both USC and SCCHWA) when necessary. An iterative process will be used with interactive strategies similar to the community charrettes, whereby poster paper notes become new primary data sources, along with field notes taken during the course of each meeting.

## Discussion

The COVID-19 challenges, responses, and resilience among Black women and their families in rural communities in southern states are critical issues for addressing health disparities and improving population wellness. Aiming to explore lived experience and resilience resources among Black women in rural areas, our study has several strengths in terms of theories, data integration, and research approach. First, the integration of multilevel resilience emphasized in the proposed study will address potential limitations or even hazards of an “individual resilience only” approach (e.g., lack of cultural reflection regarding individualism, victim blaming) and inform effective strategies to equip Black women in rural areas with supportive systems from their communities for boosting resilience. Second, the multiple sources of data collected from key stakeholders (e.g., Black women, community leaders, and community health workers) will delineate a full picture regarding individual, institutional, and social/cultural factors influencing the manifestations and effects of different resilience in the context of the southern states. Our final recommendation report based on data triangulation will inform a comprehensive, concrete, and evidence-based strategies and/or interventions tailored for Black women living in rural areas. Finally, the application of community-based participatory approach will contribute to research/operational capacity-building to paraprofessionals and local health organizations, which will, in turn, enhance resilience, increase access to care, improve public health emergency response, and address the healthcare needs of underserved subpopulations and communities affected by COVID-19 (including long COVID).

This study also has some limitations. First, the participants recruited in the study may not be representative for all the Black women in SC given not all of them can access to the recruitment flyers or have the time to receive the interview or finish the survey questionnaire. With the assistance of the community health workers rooted in the local communities, the research team will advertise our project recruitment via multiple channels and optimize their social network in reaching out the “hidden” group. Second, it is difficult to avoid bias in data collection. For example, the recall bias and socially desirable bias may occur in our in-depth interviews and self-report-based survey. Therefore, the insights and advice from the CAB through each step of the research development, implementation, and interpretation of the findings is critical and helpful. Third, the study sites are not randomly selected from all the Black rural counties in SC with a relatively small sample size. Therefore, the findings may not be generalized to the SC and beyond. Further studies with a larger sample size using random sampling are needed to improve the external validity of the resilience study among Black women in rural areas. Last, the study will not recruit Black men in rural counties therefore there is no “control group” in data analysis. This study design is based on the research aims with a focus on Black women's lived experiences. Future studies can investigate and compare the challenges and resilience by gender.

Despite these limitations, this study will have strong and sustainable public health implications in terms of improving emergency responses and informing capacity-building strategies. Through reviewing our reports, the health officers will get a comprehensive picture of the lived experience, vulnerability and resilience of rural Black women, their families, and local communities; obtain solid, multi-level, and multi-type evidence of the common challenges and typical situations Black women and their families have to face in public health crisis; and develop effective strategies and plans for resource allocations to increase the preparedness of the whole health system for future local or national emergencies.

Identifying potential resilience resources in local communities that may mitigate negative impacts of COVID-19 pandemic will inform capacity building within rural healthcare system. The findings of this proposed study will assist the state agencies and health systems in their efforts in assessing, integrating, and fostering multilevel resilience resources, particularly at the institutional and community levels. In the future, the research team will work with SCCHWA and key stakeholders through a series of meetings and workshops to finalize the assessment tools and develop training materials beyond the toolkit to assist with institutional resilience development and improvement within community health workers. Through long-term ownership by SCCHWA of the assessment instruments and training package, the resilience development will be sustained and incorporated into the capacity building efforts in response to public health emergency now and in the future.

The data collection, analysis, and interpretation will strengthen our collaboration with SCCHWA and other key stakeholders in rural communities. The dissemination of findings will further enhance the academic-government partnership in response to COVID-19 and future public health emergencies and address unique health needs among rural Black women and their families. The key stakeholders will be encouraged to share their lessons and experiences from front-line practice and give insights into their expectations and recommendations so that the research team can collectively develop plans and strategies for building a resilient health system.

In conclusion, findings in the proposed study will provide valuable references in terms of addressing SDOH challenges during the pandemic, fostering resilience, and informing evidence-based decision-making for policymakers. The study will contribute to the development of public health emergency preparedness plans, which can promote the resilience of women, their families, and local communities as well as optimize effective preparedness and response of health systems for rural Black women and their families during infectious disease outbreaks and other public health emergencies.

## Ethics statement

The studies involving human participants were reviewed and approved by the Institutional Review Boards at the University of South Carolina (Pro00123957). Informed consent will be provided by participants in the focus group discussions, in-depth interviews, and survey. All methods will be conducted in accordance with relevant guidelines and regulations. The patients/participants provided their written informed consent to participate in this study.

## Author contributions

SQ and XL conceptualized and designed the study. SQ wrote the first draft. SW and XL participated in reviewing and editing the original proposal. BO reached out and engaged local partners and community organization. SQ and SW secured the funding. All authors critically reviewed and edited the manuscript. All authors contributed to the article and approved the submitted version.
